# Compartment-specific soluble immune profiles associated with preterm birth, perinatal death, and low birthweight in pregnant individuals living with HIV

**DOI:** 10.1038/s41598-026-75643-5

**Published:** 2026-10-12

**Authors:** Jacqueline Corry, Natalia Zotova, Martine Tabala, Fidéle Lumande Kasindi, Bienvenu Lebwaze Massamba, Pelagie Babakazo, Jennifer A. Manuzak, Namal P.M. Liyanage, Nicholas T. Funderburg, Marcel Yotebieng, Jesse J. Kwiek

**Affiliations:** 1 Department of Microbiology, The Ohio State University, OH 43210 Columbus, USA; 2 Infectious Diseases Institute, The Ohio State University, OH 43210 Columbus, USA; 3 Division of General Internal Medicine, Department of Medicine, Albert Einstein College of Medicine, Bronx, NY USA; 4 School of Public Health, University of Kinshasa, Democratic Republic of the Congo, Kinshasa, Congo; 5 Department of Pathology, University of Kinshasa, Democratic Republic of the Congo, Kinshasa, Congo; 6 Center for Retrovirus Research, The Ohio State University, OH 43210 Columbus, USA; 7 Division of Immunology, Tulane National Biomedical Research Center, Covington, LA USA; 8 Division of Microbiology and Immunology, Tulane University School of Medicine, New Orleans, LA USA; 9 Department of Tropical Medicine and Infectious Disease, Tulane University Celia Scott Weatherhead School of Public Health and Tropical Medicine, New Orleans, LA USA; 10 Department of Microbial Infection and Immunity, College of Medicine, Ohio State University, Columbus, OH USA; 11 Department of Veterinary Biosciences, College of Veterinary Medicine, Ohio State University, Columbus, OH USA; 12 Division of Medical Laboratory Science, School of Health and Rehabilitation Sciences, The Ohio State University, Columbus, OH USA

**Keywords:** Adverse birth outcomes, Perinatal mortality, Maternal-fetal interface, Placental inflammation, Immune compartmentalization, Umbilical cord plasma, Biomarkers, Diseases, Immunology, Medical research, Risk factors

## Abstract

Pregnant people living with human immunodeficiency virus (PPLWH) face elevated risks of preterm birth (PTB), perinatal death (PND), and low birthweight (LBW). While antiretroviral therapy (ART) effectively suppresses viral load, it neither prevents these adverse pregnancy outcomes nor resolves underlying inflammation. Since circulating maternal markers may not fully capture local immune dysregulation, this observational cohort study identified compartmentalized immune factors associated with adverse outcomes. We analyzed 45 immune factors in paired maternal, placental, and cord plasma from 118 ART-treated PPLWH in Kinshasa, Democratic Republic of the Congo. Among these pregnancies, 30% resulted in PTB, 9% in PND, and 8% in LBW. Compared to term births, PTB (*n* = 35) was associated with discordant spatio-temporal signaling of T-cell-associated chemokines CCL20, CXCL9, and CXCL10. PND (*n* = 10) correlated with the largest inflammatory signature and was associated with factors that signal neutrophil recruitment and activation, including CXCL1, CXCL5, MPO and S100A8/A9. LBW (*n* = 9) was associated with higher concentrations of inflammatory factors at enrollment IL-18 and S100A8/A9, alongside increased postdelivery and postpartum SAA. In ART-treated PPLWH, distinct outcomes were characterized by time- and compartment-specific immune pathways. These pathways may provide mechanistic insights into adverse birth outcomes in PPLWH and highlight potential compartment-specific biomarkers for risk stratification.

**Supplementary Information:**

The online version contains supplementary material available at https://doi.org/10.1038/s41598-026-75643-5.

## Introduction

At the end of 2024, women and girls accounted for 53% of the roughly 40.8 million people living with HIV globally^1^. The health impact on this demographic is particularly severe in sub-Saharan Africa, a region which not only accounted for about 80% of all new infections among young women and girls in 2024^1^, but which is also concurrently struggling with a disproportionate malaria burden^2^. HIV can lead to poor placental development and abnormalities^3–5^, which have been associated with adverse pregnancy outcomes (APOs)^4,6–13^, including vertical HIV transmission, PTB, LBW, stillbirth, and maternal, neonatal, and infant mortality^14–25^. The risk of poor fetal outcomes is further compounded in pregnant individuals coinfected with the malaria-causing parasite *Plasmodium*, as these parasites can accumulate within the placenta^26–30^. Although ART effectively decreases HIV viral load, and minimizes vertical transmission, ART is less effective at mitigating the chronic inflammation associated with HIV infection^31–36^ and preventing HIV-associated APOs^17,24,37–44^.

In non-pregnant individuals, HIV affects the immune system in a variety of ways, eventually leading to chronic inflammation^31–33,45^. Pregnancy, however, requires precise immune regulation and timing to maintain the delicate balance necessary to sustain the fetal allograft, while maintaining robust defenses against pathogens^46–50^. For example, implantation requires localized levels of inflammatory chemokines and cytokines like growth-regulated alpha protein (CXCL1), C-X-C motif chemokine 10 (CXCL10), interleukin-6 (IL-6), IL-8, IL-15, osteopontin (SPP1), and tumor necrosis factor (TNF)^51,52^. Post-implantation, CXCL9 and CXCL10 levels decrease and IL-15 and IL-10 are secreted^47^. Natural killer cells and macrophages are converted into decidual phenotypes with reduced cytotoxicity and increased angiogenic potential^47^. Matrix metalloprotease 9 (MMP-9) and 72 kDa type IV collagenase (MMP-2) secretion allow for extracellular matrix remodeling so that placental trophoblasts can invade and anchor in the decidua^53,54^. Vascular endothelial growth factor a, long form (L-VEGF), IL-8, CXCL9, and CXCL10 are secreted and work in concert to promote trophoblast invasion and spiral artery remodeling^54^. Throughout the second and much of the third trimester IL-10 and other anti-inflammatory cytokines and regulatory cells inhibit pro-inflammatory cytokines at the maternal-fetal interface^52^. At parturition, neutrophils and macrophages are recruited to the myometrium, IL-1β is upregulated for contraction during labor, and upregulated MMP-9 reduces cervical rigidity^47,55^. Finally, in maternal blood there are dynamic changes in immune cells throughout pregnancy and the postpartum period^50^, but there are also changes in some immune factors, while others remain constant^56–59^.

When systemic infections like HIV or *Plasmodium* alter this finely choreographed immune response or misdirect immune cell trafficking the risk of APOs increases^12,47,49,52,55,60^. However, fully understanding this disruption requires a spatio-temporal approach; previous studies characterizing APOs in PPLWH have primarily focused on a single outcome of interest, measured only a small number of immune factors, or failed to evaluate plasma across multiple critical time points and anatomical compartments. To address these gaps, in this pilot study we comprehensively tested the concentrations of 45 immune factors in the blood of PPLWH on ART across multiple stages: during pregnancy, postdelivery, and postpartum. We also assessed these factors in umbilical cord blood, which represents the fetal compartment, and placental blood, which represents primarily maternal blood enriched with placenta-secreted factors. With these distinct plasma samples, we sought to determine how immune factor concentrations differ between term and PTB, live births and PND, and normal birthweight and LBW.

**Fig. 1:**
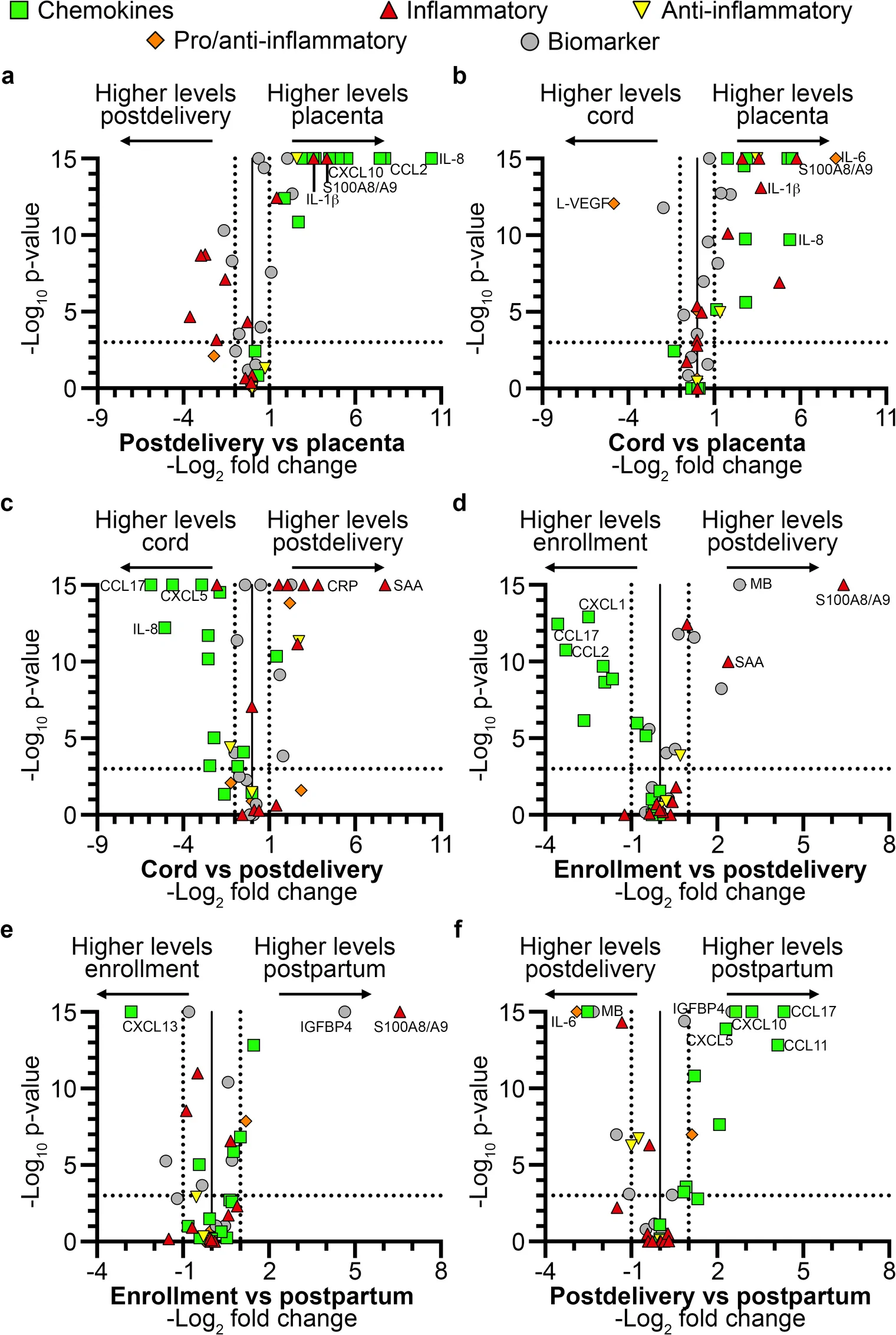
Volcano plots showing differential expression of immune factors in plasma from different locations. Differential expression plots displaying the -log_10_ of the p-value of and -log_2_ magnitude fold change in immune factors between (**a**) postdelivery and placental plasma, (**b**) cord and placental plasma, (**c**) cord and postdelivery plasma, (**d**) enrollment and postdelivery plasma, (**e**) enrollment and postpartum plasma, or (**f**) postdelivery and postpartum plasma. Statistical significance was tested by Kruskal-Wallis with a Dunn’s correction for multiple comparison. Vertical dotted lines are equivalent to a 2-fold change, horizontal dotted lines are set to a p-value that is equivalent to 0.001. Immune factor groups are defined in Supplementary Table S1.

**Fig. 2:**
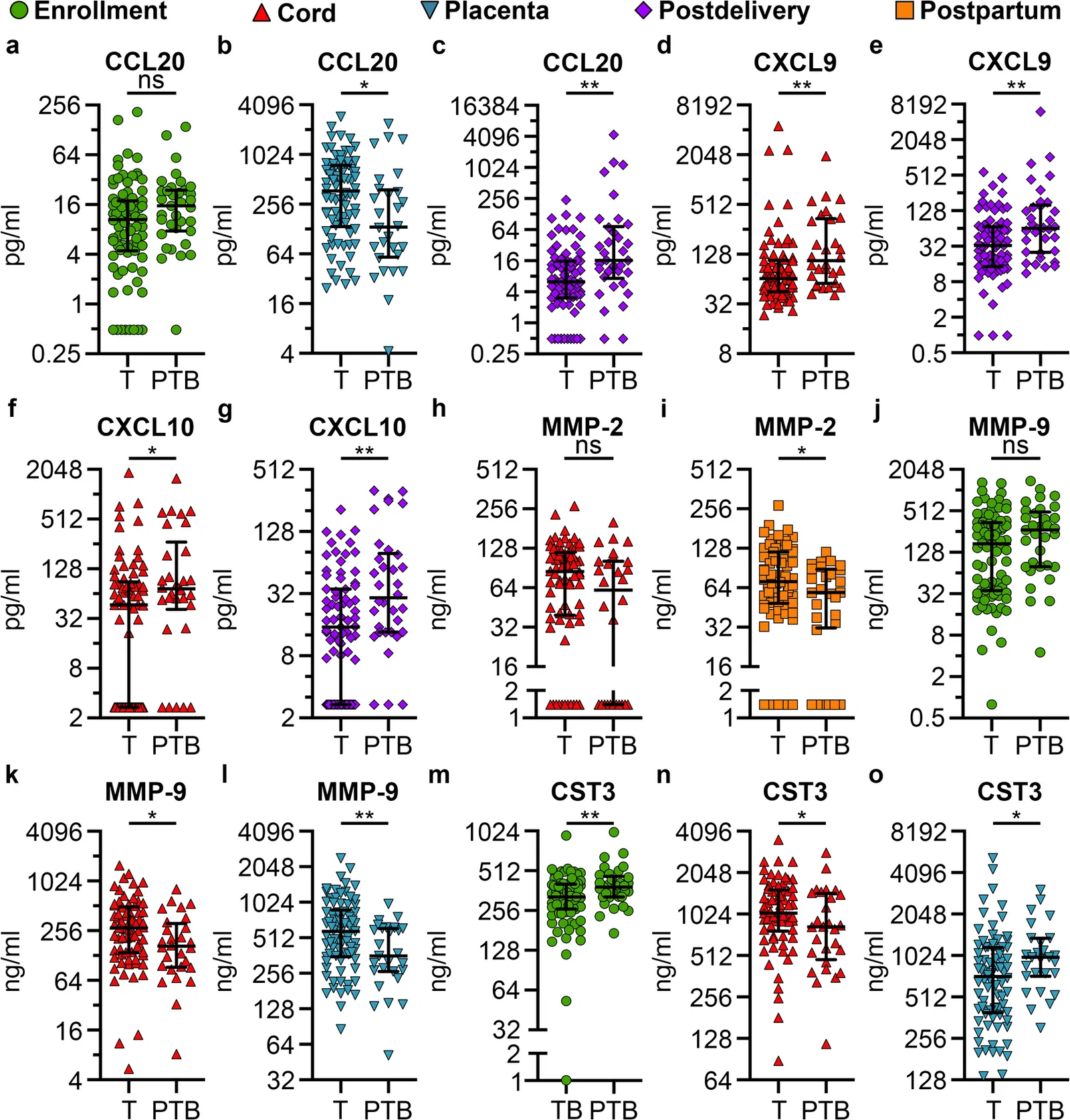
Immune factors associated with preterm birth. Immune factor concentrations were measured in plasma collected from participants at enrollment, postdelivery, and postpartum timepoints, as well as from placenta and cord blood. Concentrations were compared between individuals who delivered at term (T, *n* = 83) and from preterm births (PTB, *n* = 35). Each point represents an individual participant’s analyte concentration; horizontal lines indicate the median and IQR. Statistical significance was assessed using a two-tailed Mann–Whitney test. *p < 0.05, **p < 0.01, “ns” signifies 0.05 < *p* < 0.1. See Supplementary Table S5 for descriptive statistics.

**Fig. 3:**
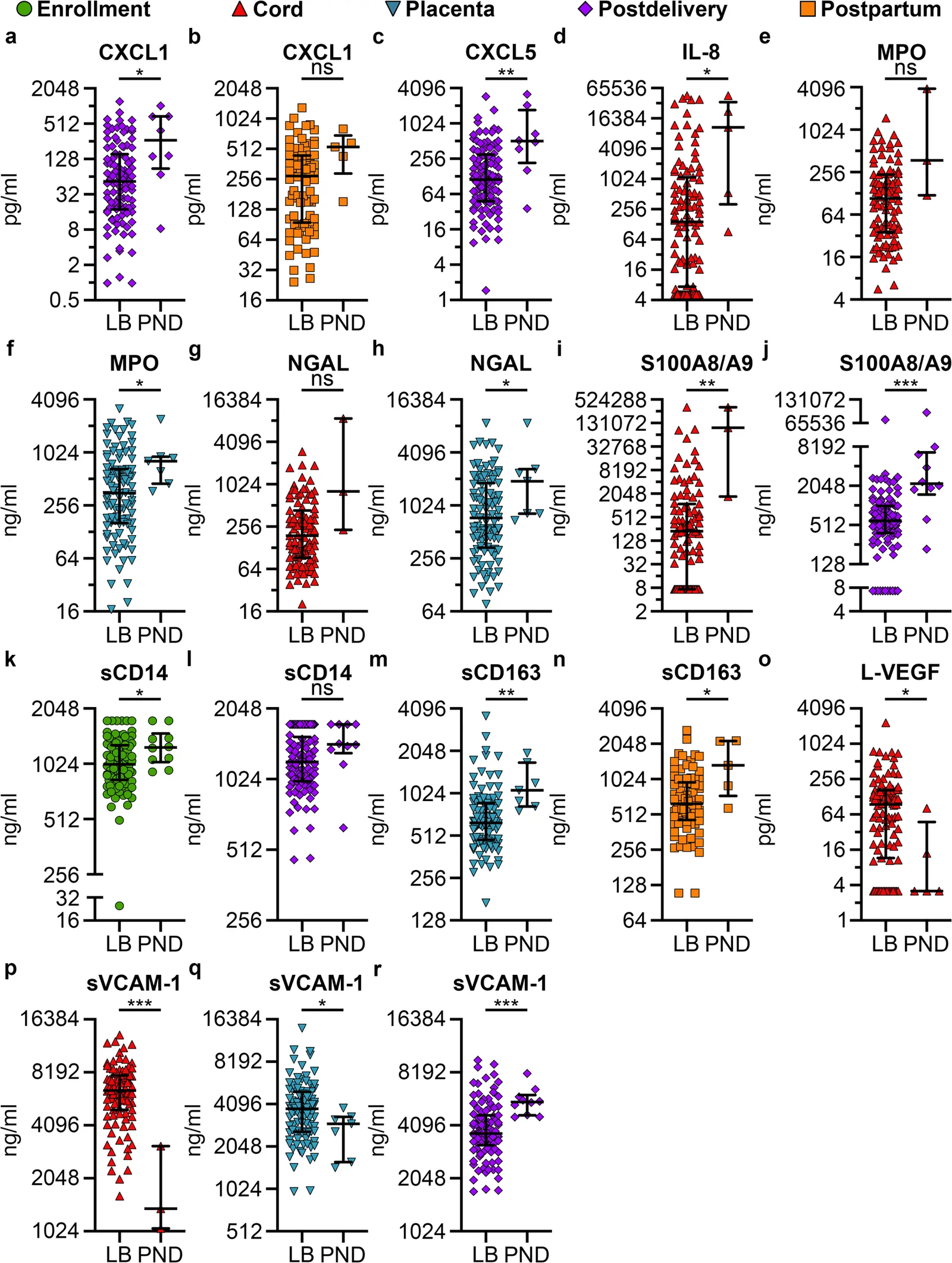
Immune factors associated with perinatal death. Immune factor concentrations were measured in plasma collected from participants at enrollment, postdelivery, and postpartum timepoints, as well as from placenta and cord blood. Concentrations were compared between live births (LB, *n* = 108) and perinatal deaths (PND, *n* = 10). Each point represents an individual participant’s analyte concentration; horizontal lines indicate the median and IQR. Statistical significance was assessed using a two-tailed Mann–Whitney test. *p < 0.05, **p < 0.01, ***p < 0.001, “ns” signifies 0.05 < *p* < 0.1. See Supplementary Table S6 for descriptive statistics.

**Fig. 4:**
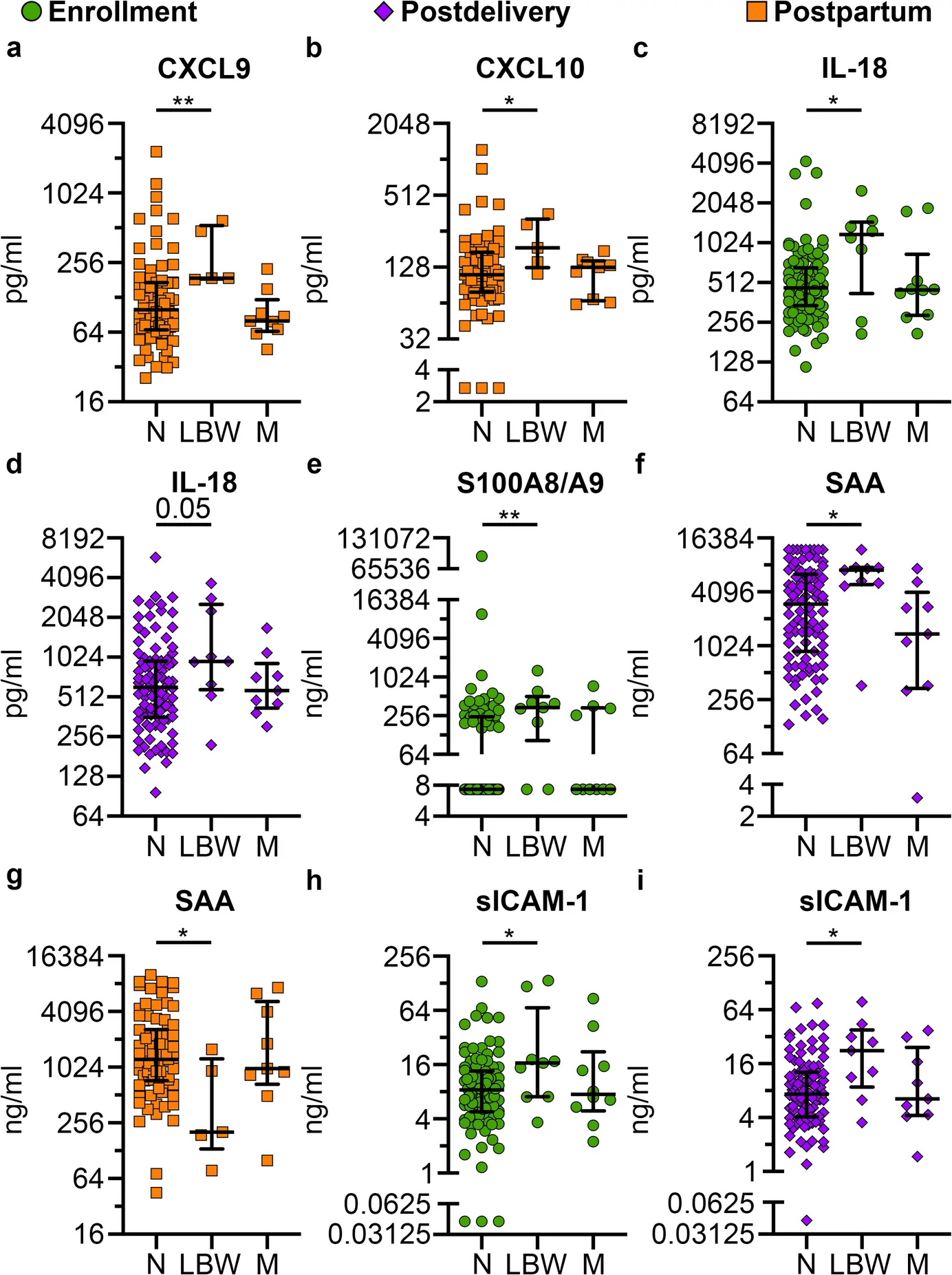
Immune factors associated with low birthweight. Immune factor concentrations were measured in plasma collected from participants at enrollment, postdelivery, and postpartum timepoints. Concentrations were compared between individuals with normal birthweight (N, *n* = 99), low birthweight (LBW, *n* = 9), and those with missing birthweight data (M, *n* = 10). Each point represents an individual participant’s analyte concentration; horizontal lines indicate the median and IQR. Statistical significance tested by Kruskal-Wallis uncorrected for multiple comparison. *p < 0.05, **p < 0.01, p-values between 0.05 and 0.1 labeled. No statistically significant difference was found between N and M. See Supplementary Table S7 for descriptive statistics.

**Fig. 5:**
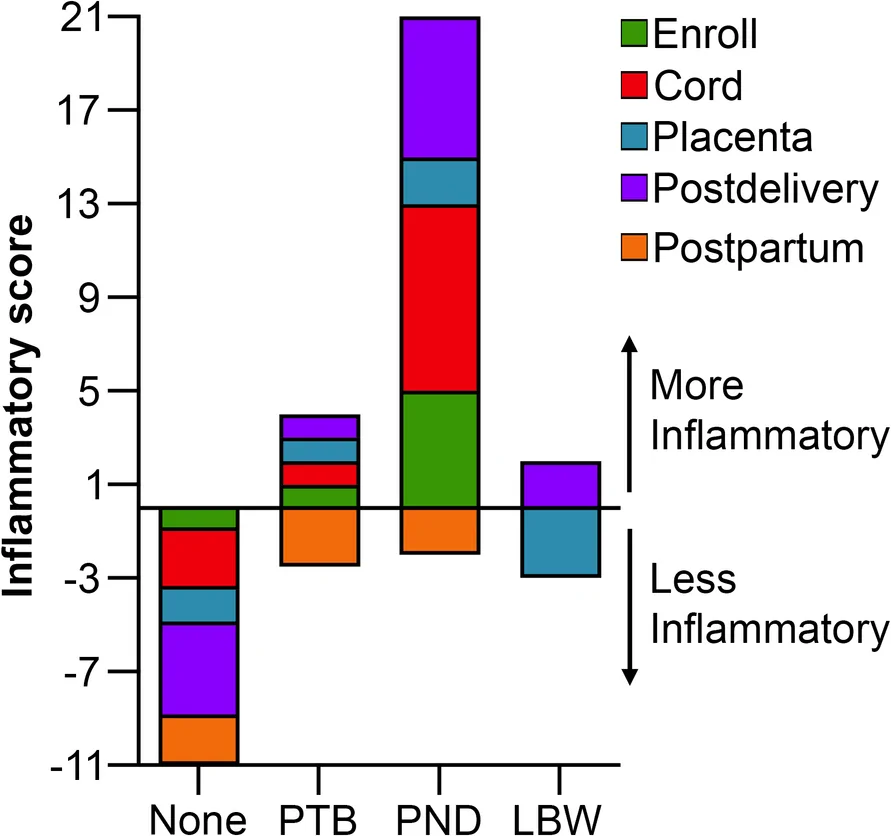
Composite inflammatory score across birth outcomes. Median inflammatory scores for no known adverse outcome (None), preterm birth (PTB), perinatal death (PND), and low birthweight (LBW), summed across plasma types. Scores were derived from 26 pro-inflammatory and 2 anti-inflammatory factors using quartile-based calculations (Supplementary Table S3-S4). Positive values indicate a more inflammatory profile. Participants can belong to multiple groups; individuals missing birthweight with no other adverse outcome were excluded.

## Results

### Participant characteristics

We enrolled 118 PPLWH; with a gestational age of 28.8 weeks (IQR: 24.4–33.5) at enrollment and 38.6 weeks (IQR: 36.0-40.1) at delivery. Maternal age was 33 years (IQR: 27–36). Most participants [94 (80%)] were multigravida, and nearly half were multiparous [58 (49%)]. The median number of pregnancies was three (IQR: 2–5). Past pregnancy complications were common, 41 (35%) had previously experienced a death *in utero*, while 24 (20%) had experienced the death of a neonate, child or infant. Only 25 PPLWH had a detectable HIV viral load at delivery, and of those, 10 had a viral load below 1000 HIV RNA copies/ml. Additionally, most participants showed evidence of past or present placental malaria infection. There were 35 (30%) neonates delivered prematurely, including one stillborn fetus, one neonatal death, one LBW neonate, and five neonates missing birthweight data. Across the entire cohort, there were ten (9%) PNDs and nine (8%) LBW neonates. (Table 1). Term and PTB cases varied by both date of enrollment (*p* = 0.003) and birthweight (*p* = 0.02). Finally, the mode of delivery differed between PND cases and live births (*p* = 0.003) (Table 2).

### Plasma immune factor concentrations differ by anatomical location

To analyze concentration differences across placental, cord and postdelivery plasma, we grouped the 45 measured immune factors into four categories: chemokines, inflammatory cytokines, pro-/anti-inflammatory cytokines, or biomarkers (Supplementary Table S1). Chemokine concentrations were highest in placental plasma: 13 of the 14 chemokines were significantly higher than in postdelivery plasma (Fig. 1a, Supplementary Fig. S1), and 9 of the 14 were significantly higher than in cord blood (Fig. 1b, Supplementary Fig. S1). Conversely, postdelivery plasma exhibited the lowest overall chemokine concentrations (Fig. 1a, c, d, f and Supplementary Fig. S1). Inflammatory markers were also higher in both postdelivery and placental plasma compared to cord blood (Fig. 1b, c and Supplementary Fig. S2). Overall, we observed greater variation in immune factor concentrations across different anatomical compartments at delivery (cord, placental, and postdelivery plasma) than was observed across different maternal time points (Fig. 1). The largest differences in chemokines between placental plasma and both the cord blood and postdelivery plasma were observed in C-C motif chemokine 2 (CCL2), CCL3, CCL4, and IL-8 (Supplementary Fig. S1, Supplementary Table S4), as well as the inflammatory markers, IL-6, Protein S100-A8/Protein S100-A9 (S100A8/A9), and serum amyloid A-1 protein (SAA) (Supplementary Fig. S2-S3, Supplementary Table S4). The differences in soluble factors were less pronounced but remained significant (Supplementary Figure S4; Supplementary Table S4). Correlation matrices were generated to visualize associations between immune factors in cord (Supplementary Fig. 5, Supplementary Table S8) and placental plasma (Supplementary Fig. S6, Supplementary Tables S9). Few negative correlations were observed. In both placental and cord blood plasma, the strongest positive correlations were among neutrophil gelatinase-associated lipocalin (NGAL), MMP-9, myeloperoxidase (MPO), and S100A8/A9 (Supplementary Fig. S5-S6, Supplementary Tables S8-S9).

### Immune factors associated with PTB

Next, we investigated whether immune factor levels were associated with PTBs. Several chemokines that have been shown to recruit T cells were associated with PTBs. CCL20 concentrations were higher at enrollment (*p* = 0.05, 1.5-fold) and postdelivery (*p* = 0.001, 2.6-fold) in PPLWH who gave birth prematurely, but were significantly lower in the placental plasma (*p* = 0.02, 0.36-fold) of PTBs compared to term births. Conversely, both CXCL9 and CXCL10 concentrations were significantly higher in the cord blood (*p* < 0.02, > 1.5-fold) and postdelivery plasma (*p* < 0.003, > 1.9-fold) of PTBs (Fig. 2, Supplementary Table S5). Furthermore, gestational age at delivery significantly negatively correlated with CCL20 and CXCL9 (r_s_: <-0.2, *p* < 0.05) concentrations across various plasma types (Supplementary Fig. S7). MMP-2 was reduced in cord (*p* = 0.09, 0.72-fold) and postpartum plasma (*p* = 0.04, 0.83-fold) of PTBs versus term births. At enrollment, both MMP-9 (*p* = 0.08, 1.6-fold) and cystatin-c (CST3) (*p* = 0.007, 1.2-fold) were higher in PTBs; however, both markers were lower in cord blood (*p* < 0.05, < 0.79-fold), in placental plasma, these markers diverged: MMP-9 was lower (*p* = 0.007, 0.62), whereas CST3 was higher (*p* = 0.02, 1.4-fold) in PTB (Fig. 2, Supplementary Table S5). All three protease-related markers significantly correlated with gestational age at delivery in at least one plasma type (Supplementary Fig. S7). IL-1RN (*p* = 0.03, 1.4-fold) NGAL (*p* = 0.03, 4.4-fold), and soluble tumor necrosis factor receptor superfamily member 1 A (sTNFRSF1A) (*p* = 0.04, 1.3-fold) concentrations were higher at enrollment in PPLWH experiencing PTB versus term births. Myoglobin (MB), a marker of skeletal and cardiac muscle damage, was lower in the postdelivery plasma (*p* = 0.02, 0.57-fold) of PTBs (Supplementary Fig. S8, Supplementary Table S5). Because participants were enrolled at different times during their pregnancy, we performed a sensitivity analysis to determine if the timing of enrollment and corresponding plasma collection affected associations between PTB and immune factors. There were no significant differences between the levels of CST3 (*p* = 0.6, 1.0-fold) or CCL20 (*p* = 0.1, 0.77-fold) by trimester; however, MMP-9 (*p* = 0.01, 0.53-fold) decreased significantly from the second to third trimester. PPLWH that enrolled in the second trimester and gave birth prematurely had significantly higher concentrations of CST3 (*p* = 0.01), this effect was not observed in PPLWH who enrolled in the third trimester (*p* = 0.2) (Supplementary Fig. S9).

### Immune factors associated with PND

Neutrophil-recruiting chemokines were generally higher in PND cases compared to live births. Specifically, CXCL1 was higher in both postdelivery (*p* = 0.02, 5.1-fold) and postpartum plasma (*p* = 0.06, 2.9-fold); CXCL5 was higher only in postdelivery plasma (*p* = 0.007, 4.5-fold); and IL-8 (*p* = 0.02, 75-fold) was higher in cord blood, although the data spread for IL-8 was wide. Markers of neutrophil and macrophage activation followed a similar pattern. MPO and NGAL concentrations were higher in the cord (*p* < 0.09, > 3.5-fold) and placental plasma (*p* < 0.05, > 2.3-fold) of PND cases, while S100A8/A9 was higher in both cord blood (*p* = 0.003, 440-fold) and postdelivery plasma (*p* = 0.0003, 3.7-fold). Furthermore, soluble monocyte differentiation antigen CD14 (sCD14) was significantly higher in PND cases only at enrollment (*p* = 0.03, 1.2-fold), with non-significantly higher values persisting through postdelivery (*p* = 0.07, 1.2-fold). Significantly higher concentrations of soluble scavenger receptor cysteine-rich type 1 protein M130 (sCD163) were observed in placental (*p* = 0.002, 1.7-fold) and postpartum plasma (*p* = 0.02, 2.1-fold). Dysregulation was also evident in L-VEGF and soluble vascular cell adhesion protein 1 (sVCAM-1), which serve as markers for endothelial activation and inflammation. Both markers were lower in cord blood (*p* < 0.003, < 0.2-fold), whereas sVCAM-1 was lower in placental plasma (*p* = 0.04, 0.78-fold) and higher in postdelivery plasma (*p* = 0.0002, 1.5-fold) in PND cases versus live births. (Fig. 3, Supplementary Table S6). Additionally, IL-1RN and MB were higher in the cord blood (*p* < 0.04, > 3.8-fold and placental plasma (*p* < 0.06, > 3.0-fold) of PND cases (Supplementary Fig. S10, Supplementary Table S6). Trimester-specific analysis revealed that while eotaxin (CCL11) (*p* = 0.09) and CCL20 (*p* = 0.1) did not vary significantly across trimesters, both were significantly higher in PND cases enrolled during the third trimester (*p* < 0.04). Conversely, CXCL9 (*p* = 0.04) and IGFBP4 (*p* < 0.0001) varied significantly by trimester. CXCL9 remained significantly associated with PND in third-trimester enrollment samples (*p* = 0.02), and IGFBP4 showed a significant association with PND in the second trimester (*p* = 0.04), but not the third (*p* = 1.0). Importantly, sCD14 did not remain significant at enrollment when analyzed by trimester (2nd : *p* = 0.3, 3rd : *p* = 0.07) (Supplementary Fig. S11).

### Immune factors associated with LBW

LBW was associated with distinct inflammatory cytokine and chemokine profiles. CXCL9 (*p* = 0.01, 1.9-fold) and CXCL10 (0.04, 1.7-fold) were higher in the postpartum plasma of LBW cases. At enrollment, plasma concentrations of IL-18 (*p* = 0.04, 2.5-fold) and S100A8/A9 (*p* = 0.005, 47-fold) were higher in LBW cases. Meanwhile, SAA concentration was more dynamic in LBW cases; it was higher in postdelivery (*p* = 0.049, 2.4-fold) and lower in postpartum plasma (*p* = 0.03, 0.2-fold). Furthermore, soluble intercellular adhesion molecule 1 (sICAM-1) exhibited a more sustained association with LBW. Concentrations were not only higher at enrollment (*p* = 0.049, 2.0-fold) and postdelivery (*p* = 0.02, 3.1-fold) (Fig. 4, Supplementary Table S7), but also negatively correlated with birthweight in placental (r_s_ = -0.30, *p* < 0.01) and postdelivery (r_s_ = -0.22, *p* < 0.05) plasma (Supplementary Fig. S13). No factors in cord blood or placental plasma were significantly associated with LBW, although MB was lower in cord blood of LBW cases (*p* = 0.098, 0.61-fold). Trimester-specific analysis revealed that both IL-18 and S100A8/A9 were significantly higher in PPLWH who enrolled in the third trimester (*p* = 0.01) and subsequently gave birth to a LBW neonate; however, neither was significantly associated with LBW in the second trimester (IL-18: *p* = 1.0, S100A8/A9: *p* = 0.06). None of the remaining factors (CXCL13, MB, sICAM-1, SPP1) retained statistical significance in this analysis (Supplementary Fig. S14).

### Composite inflammatory score across birth outcomes

Compared to the no adverse outcome group, the composite inflammatory score was higher for both PTB and PND. PND was associated with the highest overall inflammatory score, which was strongly driven by immune factors present in enrollment, cord, and postdelivery plasmas. While both PTB and PND groups exhibited positive inflammatory drivers across most plasma types, postpartum plasma acted primarily as a negative driver in these two groups, as well as in the no adverse outcome group (Fig. 5). In contrast, LBW exhibited a much smaller inflammatory footprint with fewer significantly associated immune factors. The LBW score was driven positively by postdelivery plasma and negatively by placental plasma. The negative contribution from postpartum plasma observed in other groups was completely absent in LBW cases (Fig. 5).

To assess the robustness of our composite inflammatory score which used upper and lower quartiles, a sensitivity analysis was performed utilizing the 10th and 90th percentiles. The overarching inflammatory score trends in PND remained consistent, although the magnitude of the responses was lower. The shape and magnitude of responses for no adverse outcome were similar. However, the inflammatory score of the LBW group shifted; specifically, the immune factors in postpartum and cord plasmas emerged as negative regulators, while immune factors in enrollment plasma emerged as positive regulators (Supplementary Fig. S15).

## Discussion

Our pilot study of pregnant people living with HIV revealed several key findings regarding the maternal-fetal immune environment at delivery. Overall immune factor concentrations were higher in placental plasma compared to cord blood or postdelivery plasma, despite all samples being collected at or near delivery. When examining APOs, PTB was associated with dysregulated chemokines and biomarkers most frequently rather than traditional inflammatory factors. Specifically, PTB was associated with higher levels of the T-cell recruiting chemokine CCL20. In individuals living with HIV, levels of this chemokine correlate with numbers of Th17 cells in the periphery, unbalancing the ratio of Th17 to Tregs^34^. An imbalance of Th17/Tregs is a risk factor of spontaneous preterm labor^61^. We also identified a distinct subset of immune factors including: CXCL9, IL-1RN, MB, NGAL, S100A8/A9 and sCD163 that were not associated with a particular APO but rather served as shared markers of multiple APOs. Interestingly, these demonstrate dysregulation across various compartments and time points. Finally, while the composite inflammatory score was higher for all APOs relative to individuals with no known adverse outcome, the overall level of inflammation remained lower than expected—with the notable exception of PND cases. These cases exhibited the greatest inflammatory burden driven primarily by enrollment, postdelivery and cord blood plasma. Taken together, these findings highlight that both anatomical and temporal distribution of immune factors matters just as much as their overall concentrations.

To understand why placental plasma showed such a distinct and highly concentrated immune factor profile, we first considered its unique biological context. Placental and cord blood plasma are rarely utilized in pregnancy research compared to maternal plasma^8,62–66^, likely due to collection challenges and limited collection windows in humans. Placental blood collection can be achieved through various methods, yielding different admixtures of fetal and maternal blood. The method used in this study has previously been shown to produce primarily maternal blood from the intervillous space^67^. However, as the maternal blood passes by the placenta it is enriched for factors secreted by the placenta itself. Previous studies have demonstrated that isolated placental trophoblasts and placental explants secrete immune factors in vitro and ex vivo^68–72^. Additionally, the placenta produces hormones, including immunomodulatory hormones such as cortisol^73–76^. This complexity makes it difficult to distinguish immune factors that are maternally derived from those secreted by the placenta or induced in response to placental hormones, particularly across the dynamic transition from parturition to postpartum^74–76^. Furthermore, few studies have examined changes in immune factor profiles from onset of labor through the early postpartum period^77–80^, further complicating interpretation. Our longitudinal data shows that most chemokines, including CCL2 and CCL17 decreased from enrollment to postdelivery^81^, yet remained higher in the placental plasma at delivery. In contrast, chemokines such as CCL3 remain low in maternal circulation at both enrollment and postdelivery time points^81^, but are higher in placental plasma, indicating at least transient placental expression at delivery. Taken altogether, these in vitro, ex vivo and in vivo findings suggest that the placenta may secrete certain immune factors, including chemokines, and that these may end up in maternal circulation during pregnancy. Given the central role in fetal development and the essential functions it performs^82^, the placenta has long been recognized as an important determinant in adverse fetal outcomes^3,11,12,83^. In rare cases, an abnormal placenta can even lead to maternal death^83^. Our data raise the possibility that placental dysregulation may also have subtler effects on maternal physiology through immune signaling into the maternal circulation.

In our cohort, the primary drivers of this placental dysregulation were likely infectious. Both HIV and *Plasmodium* infections can cause placental dysfunction, acute and chronic placental inflammation^3–5,84^. Because all participants were living with HIV, we were unable to directly assess the contribution of HIV itself; however, analysis of immune factors in placental plasma showed no significant differences between those with a detectable and undetectable viral load. Similarly assessing whether placental malaria contributed to immune factor levels was limited by the small number of placentas without evidence of infection. Nevertheless, three immune factors correlated with placental malaria: interferon lambda-1 (IFN-λ-1), sCD14, and MB (Supplementary Fig. S16). Previous data has demonstrated constitutive expression of IFN-λ-1 from placental epithelial cells^68,71^, making its unexpectedly low levels in our study notable. A previous study demonstrated a decrease in IFN-λ-2 in response to chronic malarial infection during pregnancy^85^ suggesting placental malarial infection may similarly suppress IFN-λ-1 expression. Since, IFN-λ-1 is generally considered immunomodulatory^86^, its reduced expression may permit an overall increase in inflammation.

The near-universal presence of placental malaria compounded this HIV-associated inflammation and limited our baseline immune comparisons. This high prevalence is perhaps less surprising given the regional context and time frame of our study. The World Health Organization reports that the Democratic Republic of the Congo accounted for 12% of the world’s reported malaria cases in 2021, second only to Nigeria^87^. Previous studies in African populations have reported high subregional prevalence alongside lower pooled regional rates^88^. Our cohort enrolled from October 2020 to May 2021; notably, the Democratic Republic of the Congo experienced a decrease in testing during this period due to the ongoing COVID-19 pandemic^89,90^, which may have resulted in underdiagnosis and undertreatment, potentially allowing ongoing transmission to go undetected in the broader community. While earlier research indicates that individuals in their first or second pregnancies are most susceptible to placental malaria^28–30^, a number of studies in pregnancies affected by HIV without ART suggest that gravidity is less of a factor in placental invasion in the context of HIV-*Plasmodium* coinfection^88,91,92^. Although PPLWH in our study were on ART and we had a large number of multigravida individuals, our placental pathologists diagnosed all but two available placentas as having evidence of current or past *Plasmodium* invasion of the placenta. Correspondingly, we observed a large number of stillbirths in our study (7% versus a national average of 2.6%^93^ and PTBs (30% versus a national average of 12.4%^94^. While HIV can increase the risk of both stillbirth and PTB^14,15,17,19,24,25^, *Plasmodium* elevates these risks as well^24,26,95–97^, particularly if parasites infect the placenta^26,84,96,97^. Consequently, this exceptionally large burden of placental malaria could help to explain the observed increases in stillbirths and PTB within our cohort.

While malaria and HIV likely contributed to specific APOs, PND more broadly remains a critical yet understudied phenomenon^98–101^, partly due to inconsistent terminology^102–104^ and psychosocial stigma that may lead to underreporting^99,100,105^. Known risk factors include: infection, placental and umbilical cord pathology, birthweight, gestational age at delivery, and maternal history/health^11,18,19,102,106–108^. PND cases in our cohort primarily exhibited higher concentrations of chemokines linked to T cell, monocyte and granulocyte recruitment^109^, alongside, heightened markers of neutrophil and macrophage activation^110–114^. Although neutrophils are typically scarce in healthy placental tissue^12^, both neutrophils and macrophages have been associated with PND in placental, cord, and/or fetal tissues, particularly in the context of fetal infection^3,12,13,115^.

This intense innate immune activation in PND cases was further evidenced by increases in S100A8/A9, MPO, NGAL and MMP-9, primary components of human neutrophil extracellular traps (NETs)^116^. NETs are networks of decondensed chromatin and intracellular proteins extruded from activated neutrophils^116^, although some of these immune factors can be secreted by macrophages as well^110,117^. The broader elevation of NET-associated proteins points to intense innate immune activation. In concordance with our results, Sarr et al. observed that although peripheral neutrophil counts were lower in PPLWH with malaria than in those who were uninfected, there were increased markers of neutrophil activation in the placenta and placental blood^118^. Although neutrophils serve critical homeostatic and protective functions throughout pregnancy, their activation and NET release must be carefully balanced^49,55^ to prevent generating a severely inflammatory and cytotoxic microenvironment. Specifically, S100A8/A9 is a damage-associated molecular pattern (DAMP) that triggers local inflammation^111^; MPO catalyzes hypochlorous acid production, driving inflammation by activating redox-sensitive cascades and inducing cellular damage^119^; and NGAL forms complexes that prevent the degradation of MMP-9, thereby facilitating extracellular matrix breakdown, immune cell migration, and dysregulated cytokine and chemokine activity^120^. Collectively, these findings suggest that mislocalized or untimely activation of innate immune cells, specifically monocytes, macrophages and neutrophils play an important role in the immunopathology of PND.

Beyond this localized innate cell activation, specific chemokines pointed to shared, systemic inflammatory signaling across multiple APOs. Most notable was CXCL9, a chemotactic factor primarily responsible for recruiting T cells to inflammatory sites^109^. In pregnancy, CXCL9 has been associated with villitis of unknown etiology, an inflammatory placental disease linked to PTBs, LBW, and PND^3,13,121,122^. While a previous study suggested that CXCL9 may be predictive of both pregnancy loss and preterm delivery^123^, our analysis found CXCL9 is more highly expressed in PND at enrollment and in PTB in cord blood and postdelivery plasma. Interestingly, CXCL11, which signals through the same receptor as CXCL9, was lower in the placental plasma of PND cases, an opposite trend to CXCL9. A similar phenomenon has been observed in the placentas of spontaneous versus elective abortions^124^. In vitro studies have demonstrated that CXCL11 drives T cell polarization toward a regulatory phenotype, whereas CXCL9 drives polarization toward a more inflammatory Th1 or Th17 subset^125^. Both Th1 and Th17 cells have been implicated in PTB^61,126^ while Th1 cells have been specifically implicated in PND^13^. Ultimately, CXCL9 serves as a broad marker of APOs across multiple maternal time points and anatomic locations, potentially driving pathology by recruiting T cells to the wrong site at the wrong time.

In stark contrast to the pro-inflammatory profile of CXCL9 and localized NET formation, we also observed concurrent elevations of IL-1RN. This potentially anti-inflammatory molecule competitively inhibits the pro-inflammatory cytokines IL-1α and IL-1β^127^. Because of this, multiple drugs have been developed to block IL-1 signaling^128–130^. One preclinical drug, Rytvela, significantly reduced not only PTB, but also fetal death in a mouse model of lipopolysaccharide-mediated PTB^131^. In our study, IL-1RN was consistently higher in multiple plasma types across APOs; however, we did not observe significant differences in IL-1β under any conditions and did not test IL-1α. Though *IL1B* and *IL1RN* are in close proximity on chromosome 2, and are induced by similar stimuli, they have unique transcriptional and regulatory pathways^132^. In addition, in order to release IL-1β cells need to undergo pyroptosis, an inflammatory form of cell death^133^; however, IL-1RN occurs in both a secretable and non-secretable form^134^. Taken together, the increased IL-1RN in our cohort is likely a marker of immune pathology.

While these diverse immune markers highlight the molecular pathology of PND and PTB, MB served as a clear indicator of the resulting physical tissue damage across multiple APOs. We identified MB, a marker of striated muscle injury^135,136^, as being associated with multiple APOs. MB was higher in PND cases, indicating possible skeletal or cardiac injury in the fetal compartments. This difference was observed both in stillbirths and neonatal deaths, suggesting the damage is severe, but not uniformly fatal prior to delivery. One population-based mortality surveillance found that the most common cause of stillbirths in seven countries in Africa was hypoxia^137^, which can lead to myocardial damage^138^. In contrast, PTB was associated with decreased MB levels postdelivery despite no difference in delivery mode; this decrease potentially reflects a shorter second-stage labor—the period of maximum muscle strain—which has previously been shown to be briefer in PTBs compared to term births^139,140^. Alternatively, given the rapid clearance of MB from circulation after acute injury^141^ differences in sampling timing could account for this finding.

Indeed, these challenges surrounding sampling timing point to broader limitations within our study design. While this pilot study provides valuable preliminary insights, several limitations should be noted. Methodologically, our study was conducted within a larger cohort of pregnant people living with HIV and lacked both HIV-negative and placental malaria-negative comparator group. Furthermore, as previously discussed^81^, there was temporal variability in collection of both the postdelivery (1–3 days post-birth) and enrollment timepoints (15 to 40 weeks gestational age). This wide enrollment window coincides with dynamic changes in maternal immune cell populations, blood volume and soluble factors in the blood^50^. Although current longitudinal data on maternal immune factor fluctuations remain sparse, existing studies indicate that while some immune factors remain stable, others change significantly across gestation^56–58^. Indeed, our analysis suggests that several evaluated immune factors, including CXCL9, MMP-9, and NGAL, change between the second and third trimesters. Such gestational shifts could influence the magnitude of observed associations, potentially obscuring immune factors that are specifically important early or late in pregnancy. However, further parsing or stratifying the data by gestational age was constrained by our modest sample size. Follow-up studies would benefit from standardized enrollment timelines and expanded sample sizes to fully characterize the dynamic interplay between immune factors and gestation, while remaining adaptable to local healthcare access realities.

Compounding these temporal challenges, our small size reduced our statistical power and increased potential outlier influence, particularly given the limited number of PND and LBW cases. Crucially, the subgroup analyses presented here should be viewed as exploratory and hypothesis-generating. To minimize Type II errors in our small cohort, we omitted false-discovery corrections for tests with small sample sizes. However, we acknowledge that running uncorrected analyses on a multiplex dataset of 45 analytes across multiple compartments and time points increases the risk of incorrectly rejecting the null hypothesis. Consequently, some identified immune factors may have been incorrectly implicated. Finally, our cohort is drawn from a concentrated geographic area. Given that immunologic signatures in people living with HIV and pregnant individuals are known to vary regionally^142,143^, these specific signatures may not be broadly generalizable. Ultimately, these constraints highlight a clear path forward, emphasizing the need for larger, geographically diverse cohorts with more controlled gestational time points to validate and expand upon these initial findings.

Despite these constraints, this study clearly demonstrates that pregnant people living with HIV on ART exhibit distinct, time- and compartment-specific soluble immune factor profiles that are significantly associated with APOs. By analyzing plasma in multiple anatomical compartments across the peripartum, we established that inflammation is not uniform across the fetal-maternal interface. We further demonstrated that there were both anatomical and temporal-specific immune factors significantly associated with APOs, which suggests that despite ART, APOs in PPLWH and placental malaria are driven by complex, localized immune system dysregulation. This highlights the critical need to test whether anti-inflammatory therapies could improve pregnancy outcomes among pregnant people living with HIV taking ART, particularly those that are also impacted by placental malaria.

## Methods

### Definitions

For clarity, we categorized key concepts into immunological, obstetrical, and infection-related definitions. Immunologically, cytokines are defined as peptides or proteins secreted by cells that can have autocrine, paracrine or endocrine effects on target cells that express the requisite receptor(s). Pro-inflammatory cytokines induce, or trigger inflammation, whereas anti-inflammatory cytokines decrease it. Chemokines are a subset of cytokines that induce directional movement along a gradient. Damage-associated molecular patterns (DAMPs) are intracellular molecules that are released from dead or dying cells; they trigger inflammation by binding to pattern recognition receptors to signal cellular stress or tissue injury^144^. Acute phase proteins (APPs) are a class of proteins whose concentration in blood increases in response to injury, trauma, or infection^145^. In this manuscript, “biomarkers” refers to non-cytokine molecules whose measurable blood concentrations change in response to inflammation but which neither bind cytokine receptors nor actively drive the inflammatory process. The term “immune factor” is the collective name for all analyzed cytokines, chemokines and biomarkers.

In terms of obstetrical definitions, gestational age was based on the self-reported last menstrual period at enrollment when available (*n* = 95), or clinical estimates when not (*n* = 23). Births prior to 37 weeks’ gestation were classified as preterm births (PTBs); those at or after 37 weeks as term birth. Perinatal death (PND) encompassed stillbirth (fetal death after 28 weeks but prior to or during birth^146^ and neonatal death (death within the first 28 days of date of birth). Live birth (LB) includes all neonates that survived 48 h after birth. Neonate weights at delivery were categorized as low birthweight (LBW, defined as ≤ 2500 g), normal birthweight (defined as > 2500 g), or missing for neonates lacking birthweight information. Blood draws occurred at enrollment (between gestational weeks 15 and 40), 1–3 days postdelivery (PD), and approximately 6 weeks postpartum (median 6.7 weeks; 95% CI 6–10 weeks). Cord blood refers to blood collected from the umbilical cord that represents the fetal compartment, collected at time of delivery, and placental blood—primarily maternal blood from the intervillous space—was collected immediately after the cord blood^67^.

The technical limit of detection for HIV RNA was < 40 HIV RNA copies/ml of blood, (see below for methods). Placental *Plasmodium* infection classifications were made by a pathologist and were based on Bulmer’s classification^147^: an *active infection* was characterized by the presence of parasites in maternal erythrocytes within the intervillous spaces and malarial pigment (hemozoin) in circulating erythrocytes and/or monocytes, with a notable absence of hemozoin or plasmodial cells in fibrin. An *active-chronic infection* exhibited the same characteristics as an active infection but additionally featured the presence of hemozoin or plasmodial cells in fibrin, the syncytiotrophoblasts, and/or the stroma. Finally, a *past-chronic infection* was characterized by a lack of parasites in erythrocytes alongside the presence of hemozoin pigment or plasmodial cells confined to fibrin.

### Study setting

This study was an observational cohort study nested within the larger randomized control trial for the Continuous Quality Improvement-Prevention of Mother to Child Transmission (CQI-PMCT)^148–154^. The parent study was an open label, parallel, group randomized trial designed to evaluate continuous quality improvements (CQI) on long-term ART outcomes among pregnant and breastfeeding individuals receiving care in Kinshasa province.

### Ethics approval and consent to participate

The authors confirm that the ethical policies of the journal, as noted on the journal’s author guidelines page, have been adhered to, the appropriate ethical review committee approval has been received, and all methods were performed in accordance with relevant guidelines and regulations. The study conformed to the US and the Democratic Republic of the Congo Policies for the Protection of Human Subjects. This is an observational cohort study within a larger study approved by Institutional Review Boards (IRB) at The Ohio State University (IRB study ID: 2015H0440), Albert Einstein College of Medicine (protocol #2020–12018) and the Kinshasa School of Public Health Ethics Committee (protocol #0001 1–04101-00001365292–20). Written informed consent was obtained directly from participants. Data were de-identified prior to processing and analysis.

### Study design, participants, and specimen collection

Drawing from the CQI-PMCT framework, we identified all pregnant individuals in their second or third trimester living with HIV and receiving care at any of the participating maternal and child health facilities between October 2020 and May 2021. After obtaining written consent, 199 eligible participants were enrolled. Of these, 81 were excluded from the final analysis due to the availability of only a single plasma sample (*n* = 28), missing important delivery information (e.g., birthweight, baby’s sex, mode of delivery, or pathologic information on the placenta/umbilical cord; *n* = 48), or multiple births (*n* = 5). The final cohort for this analysis consisted of 118 participants.

To monitor these participants longitudinally, trained personnel administered questionnaires and collected maternal peripheral venous blood and dried blood spots at three specific time points: enrollment, 1–3 days postdelivery, and approximately 6 weeks postpartum. Venous blood was drawn into EDTA-containing tubes, centrifuged for 10 min at 200 × g, and the resulting plasma was aliquoted and stored at -80 °C. Concurrently, dried blood spots were obtained via finger prick on Whatman 903 protein saver cards (Cytiva), dried at room temperature for at least three hours, then packaged with desiccant and stored at -20 °C. Postdelivery dried blood spots were tested for HIV viral RNA using the m2000rt Real-Time HIV-1 assay (Abbott) by the National AIDS Reference Laboratory in Kinshasa.

At delivery the umbilical cord and placenta were collected. The cord was washed with normal sterile saline and approximately 3 cm from its insertion into the placenta a 5 ml syringe was inserted into the umbilical vein with a 20–22-gauge needle was used to aspirate cord blood into an EDTA collection tube. Following cord blood collection, the placental prick method described by Othoro et al. was employed to collect blood from the intervillous space into EDTA-containing tubes^67^. Cord and placental blood were centrifuged for 10 min at 200 x g, and plasma was aliquoted and stored at -80 °C. Placentas were weighed and 2 × 1 × 0.5 cm sections were taken from the maternal and fetal surfaces as previously described^155^. The Bulmer histological classification^147^ was used to classify placental plasmodial lesions.

### Specimen processing and laboratory testing

Following sample collection in the Democratic Republic of the Congo, all plasma aliquots were shipped on dry ice to The Ohio State University (Columbus, Ohio) and stored at -80 °C. Prior to assaying, samples were thawed and centrifuged twice at 2,500 x g for 5 min to remove particulates.

Immune factor names and abbreviations were standardized via UniProt (*Homo sapiens*) following the International Protein Nomenclature Guidelines^156^; full details including units of measure and kit purchasing information are provided in Supplementary Table S1. The LEGENDplex Human Proinflammatory Chemokine Panel 1 (BioLegend, 740984), LEGENDplex COVID-19 Cytokine Storm Panel 1 & 2 (BioLegend, 741095) and LEGENDplex Vascular Inflammation panel 1 (BioLegend, 740551) were used to measure immune factors according to the manufacturer’s filter plate protocol as previously described^81^. Briefly, data were collected on the MACS Quant 10 Flow cytometer (Miltenyi Biotec) and using MacsQuant Software (Miltenyi Biotec). Data analysis was performed using the LEGENDplex cloud-based Qognit software (BioLegend). Gates established from the assay standards were applied uniformly to all samples, and analyte concentrations were interpolated using five-parameter logistic standard curves. Additionally, seven specific immune factors (CXCL13, IL-4, IFN-λ-1, sCD14, sCD163, sTNFRSF1A, and sTNFRSF1B) were individually measured via DuoSet ELISA (R&D Systems) with minor modifications^81^. Absorbances were read on a SpectraMax i3x (Molecular Devices). See Supplementary Table S2 for dilution factors, limits of detection (LoD), sample number, and samples (n and %) above and below the LoD.

### Composite inflammatory score

To quantify overall inflammation, we calculated a composite inflammatory score, by adapting a previously described method^157^ to include both the upper (> 75 percentile) and lower quartiles (< 25th percentile). For concentrations > 75th percentile, values were scored + 1 for pro-inflammatory factors and − 1 for anti-inflammatory factors, following the original framework. For concentrations < 25th percentile, values were scored − 1 for pro-inflammatory factors and + 1 for anti-inflammatory factors. Values between the 25th and 75th percentiles, missing values, and 16 mixed anti-/pro-inflammatory cytokines or general biomarkers were unscored (assigned a value of 0). Immune factors demonstrating no interquartile variance for a given plasma type, such as IFN-λ−1, were also excluded. Immune factor scoring assignments can be found in Supplementary Table S3. Scores were summed for each individual by plasma type, and the median was calculated for each APO group. Individuals could appear in multiple groups because APO categories were not mutually exclusive. A sensitivity analysis was performed using the 10th and 90th percentiles under this same expanded scoring framework (Supplementary Table S3).

### Statistical analyses

Data processing was performed in Microsoft Excel, while statistical testing and visualization were conducted in GraphPad Prism (v. 10.4.1–10.6.0). Descriptive statistics for composite inflammatory score, immune factor data, and APOs are available in Supplementary Tables S3-S7. Depending on the data type and distribution, significance was evaluated using two-tailed Mann-Whitney tests, two-sided Fisher’s exact tests, Kruskal-Wallis tests with or without Dunn’s correction for multiple comparisons, or two-tailed Spearman correlations (significance cutoffs: rs < -0.2 or > 0.2, *p* < 0.05). Given the exploratory and hypothesis-generating nature of this study, we did not uniformly correct for multiple hypothesis testing across all findings^158^. Although omitting post-hoc adjustments like Dunn’s correction increases the risk of Type I error, it minimizes Type II error, thereby preventing the premature rejection of potentially significant biological trends. To ensure rigorous reporting, uncorrected analyses are primarily reserved for supplementary data, with the exception of one main text figure comparing normal, low, and missing birthweights.

**Table 1:** Obstetrical data and outcome.

Variable	*N* (%)	Median (IQR)
Sample collection (wks)		
Enrollment		29 (24, 33)
Postdelivery		39 (36, 40)
Postpartum		6.7 (6.4, 7.3)
Maternal age		33 (27, 36)
Gravida		
Primigravida	24 (20)	
Multigravida	94 (80)	
Parity		
Nullipara	33 (28)	
Primipara	27 (23)	
Multipara	58 (49)	
Number of pregnancies	3 (2, 5)	
Previous pregnancy outcomes		
Death *in utero*		
Yes	41 (35)	1 (1, 2)
No	53 (45)	
Missing	24 (20)	
Neonate/infant/child death		
Yes	24 (20)	1 (1, 2)
No	70 (59)	
Missing	24 (20)	
HIV viral RNA		
Detectable	25 (21)	
Undetectable	93 (79)	
ART		
TDF+3TC + DTG	85 (72)	
TDF+3TC + EFV	30 (25)	
Missing	3 (3)	
Placental malaria		
None observed	2 (2)	
Active	6 (5)	
Active-chronic	17 (14)	
Past-chronic	90 (76)	
Missing	3 (3)	
Intermittent preventative therapy		
No doses	51 (43)	
1 dose	25 (21)	
2 doses	12 (10)	
3 doses	8 (7)	
Missing	22 (19)	
Preterm birth (PTB)		
No	83 (70)	
Yes	35 (30)	
Perinatal death (PND)		
Stillbirth	8 (7)	
Neonatal death	2 (2)	
Low birthweight (LBW)		
No	99 (84)	
Yes	9 (8)	
Missing	10 (8)	
Birthweight (kg)		3.0 (2.8, 3.4)
Placental weight (kg)		0.6 (0.5, 0.7)

Abbreviations: IQR, interquartile range; ART, antiretroviral therapy; TDF, tenofovir; 3TC, lamivudine; DTG, dolutegravir; EFV, efavirenz.

**Table 2:** Obstetrical data by adverse outcome☨.

Variable	T (*n* = 83)	PTB (*n* = 35)	*p*- value‡	LB (*n* = 108)	PND (*n* = 10)	*p*- value‡	*N* (*n* = 99)	LBW (*n* = 9)	M (*n* = 10)	*p*- value∝
Maternal age	32 (28, 36)	34 (28, 37)	0.24	33 (27, 36)	34 (32, 39)	0.39	33 (28, 37)	34 (26, 35)	34 (27, 37)	0.97
Sample collection										
Enrollment	30 (25, 36)	26 (22, 32)	**0.003**	29 (24, 33)	33 (27, 37)	0.1	29 (24, 34)	31 (29, 35)	25 (21, 26)	0.05
Postdelivery	39 (38, 41)	35 (33, 36)	**< 0.0001**	39 (36, 40)	38 (37 42)	0.64	39 (36, 40)	39 (39, 40)	37 (35, 38)	0.13
Postpartum	6.7 (6.4, 7.3)	6.7 (6.4, 7.0)	0.73	6.7 (6.4, 7.3)	6.6 (6.6, 7.3)	0.8	6.7 (6.4, 7.3)	6.9 (6.5, 7.0)	6.4 (6.4, 7.4)	0.96
Mode of delivery										
Cesarian	13 (16)	9 (26)	0.55	19 (18)	3 (30)	**0.003**	21 (21)	0 (0)	1 (10)	0.62
Dystocia	2 (2)	0 (0)		0 (0)	2 (20)		2 (2)	0 (0)	0 (0)	
Eutocia	67 (81)	26 (74)		88 (81)	5 (50)		75 (76)	9 (100)	9 (90)	
Missing	1 (1)	0 (0)		1 (1)	0 (0)		1 (1)	0 (0)	0 (0)	
Gravidity										
Primigravida	17 (20)	7 (20)	> 0.99	23 (21)	1 (10)	0.68	21 (21)	2 (22)	1 (10)	0.81
Multigravida	66 (80)	28 (80)		85 (79)	9 (90)		78 (79)	7 (78)	9 (90)	
HIV-viral load										
Detectable	16 (19)	9 (26)	0.47	23 (21)	2 (20)	> 0.99	20 (20)	2 (22)	3 (30)	0.73
Undetectable	67 (81)	26 (74)		85 (79)	8 (80)		79 (80)	7 (78)	7 (70)	
ART										
TDF+3TC + DTG	62 (75)	23 (66)	0.54	76 (70)	9 (90)	> 0.99	73 (74)	7 (78)	5 (50)	0.31
TDF+3TC + EFV	19 (23)	11 (31)		29 (27)	1 (10)		24 (24)	2 (22)	4 (40)	
Missing	2 (2)	1 (3)		3 (3)	0 (0)		2 (2)	0 (0)	1 (10)	
Placental malaria										
None observed	1 (1)	1 (3)	0.85	2 (2)	0 (0)	0.51	2 (2)	0 (0)	0 (0)	0.43
Active	5 (6)	1 (3)		5 (5)	1 (10)		6 (6)	0 (0)	0 (0)	
Active-chronic	11 (13)	6 (17)		17 (16)	0 (0)		17 (17)	0 (0)	0 (0)	
Past-chronic	64 (77)	26 (74)		81 (75)	9 (90)		72 (73)	8 (89)	10 (100)	
Missing	2 (2)	1 (3)		3 (3)	0 (0)		2 (2)	1 (11)	0 (0)	
Birthweight (kg)	3.1 (3.0, 3.4)	2.9 (2.8, 3.2)	**0.02**	3.1 (2.8, 3.4)	3.1 (2.9, 3.3)	0.94	3.1 (3, 3.4)	2.5 (2.4, 2.5)	N/A	**< 0.0001**
Placental weight (kg)	0.6 (0.5, 0.7)	0.5 (0.5, 0.7)	0.14	0.6 (0.5, 0.7)	0.6 (0.5, 0.7)	0.68	0.6 (0.5, 0.7)	0.5 (0.4, 0.5)	0.6 (0.5, 0.7)	0.08

Abbreviations: T, term birth; PTB, preterm birth; LB, live birth (excluding neonatal death); PND, perinatal death; N, normal birthweight; LBW, low birthweight; M missing birthweight; ART, antiretroviral therapy; TDF, tenofovir; 3TC, lamivudine; DTG, dolutegravir; EFV, efavirenz.

☨All data are median (IQR) for continuous values or absolute numbers (percentages) for categorical values.

‡ Mann-Whitney test was used to analyze the relationship between the groups with numerical values, while Fischer’s exact test was used for those without.

∝ Normal vs. low vs. missing birthweight. Kruskal-Wallis with Dunn’s correction for multiple comparisons was used to analyze the relationship between all three groups with numerical values, a Fischer exact test was used for those without, for comparison of birthweights, a Mann-Whitney test was performed.

## Supplementary Information

Below is the link to the electronic supplementary material.

Supplementary Material 1

Supplementary Material 2

## Data Availability

The summary data needed to evaluate this manuscript are in included in supplemental tables. Summary data for minor supplemental figures were not included in the manuscript and will be provided upon reasonable request.

## Abbreviations

3TC: Lamivudine
APP: Acute phase protein
APO: Adverse pregnancy outcome
ART: Antiretroviral therapy
C: Cord blood
CCL: C-C motif chemokine
CCL11: Eotaxin
CQI: Continuous quality improvement
CQI-PMCT: Continuous quality improvement-prevention of mother to child transmission
CRP: C-reactive protein
CST3: Cystatin-C
CXCL1: Growth-regulated alpha protein
CXCL: C-X-C motif chemokine
DAMP: Damage-associated molecular pattern
DTG: Dolutegravir
EFV: Efavirenz
GM-CSF: Granulocyte-macrophage colony-stimulating factor
HIV: Human immunodeficiency virus
IFN: Interferon
IGFBP4: Insulin-like growth factor binding protein
IL-: Interleukin
IQR: Interquartile range
IRB: Institutional Review Boards
L-VEGF: Vascular endothelial growth factor a, long form
LB: Live birth
LBW: Low birthweight
LoD: Limit of detection
M: Missing birthweight
MB: Myoglobin
MMP-2: 72 kDa type IV collagenase
MMP-9: Matrix metalloproteinase-9
MPO: Myeloperoxidase
N: Normal birthweight
NETs: Neutrophil extracellular traps
NGAL: Neutrophil gelatinase-associated lipocalin
P: Placental blood
PD: Postdelivery
PND: Perinatal death
PPLWH: Pregnant people living with human immunodeficiency virus
PTB: Preterm birth
S100A8/A9: Protein S100-A8/Protein S100-A9
SAA: Serum amyloid A-1 protein
sCD14: Soluble monocyte differentiation antigen CD14
sCD163: Soluble scavenger receptor cysteine-rich type 1 protein M130
sICAM-1: Soluble intercellular adhesion molecule 1
SPP1: Osteopontin
sTNFRSF1: Soluble tumor necrosis factor receptor superfamily member 1
sVCAM-1: Soluble vascular cell adhesion protein 1
T: Term birth
TDF: Tenofovir
TNF: Tumor necrosis factor
