# Supplementary material for: Compartment-specific soluble immune profiles associated with preterm birth, perinatal death, and low birthweight in pregnant individuals living with HIV
Source: Sci Rep. 2026 Oct 12;16:34647. doi: 10.1038/s41598-026-75643-5 (PMC13713567; doi:10.1038/s41598-026-75643-5)
Supplement: Supplementary file 1 [file 41598_2026_75643_MOESM2_ESM.docx]

# Compartment-specific soluble immune profiles associated with preterm birth, perinatal death, and low birthweight in pregnant individuals living with HIV

Jacqueline Corry, Natalia Zotova, Martine Tabala, Fidéle Lumande Kasindi, Bienvenu Lebwaze Massamba, Pelagie Babakazo, Jennifer A. Manuzak, Namal P.M. Liyanage, Nicholas T. Funderburg, Marcel Yotebieng, Jesse J. Kwiek

# **Supplementary figures:**

## Supplementary Figure S1. Chemokine expression in cord, placental, and postdelivery plasma. Chemokines were measured using (A-L, N) LEGENDplex assays and (M) ELISAs in plasma collected from cord (C) blood, placental (P) blood, and at the postdelivery (PD) timepoint. Each point represents an individual participant’s analyte measurement, with horizontal lines indicating the median and interquartile range (IQR). Dotted lines represent limits of detection (LoD), see Supplementary Table S2 for all LoD values and Supplementary Table S4 for descriptive statistics. Statistical significance tested by Kruskal-Wallis with a Dunn’s correction for multiple comparisons. ns=not significant, *p<0.05, **p<0.01, ***p<0.001, ****p<0.0001.

## Supplementary Figure S2. Pro/anti-inflammatory expression in cord blood, placental, and postdelivery plasma. Pro- and anti-inflammatory cytokines were measured using LEGENDplex assays in plasma collected from cord blood (C), placental (P) blood, and at the postdelivery (PD) timepoint. Immune factors were graphed in pg/ml (A-K) or ng/ml (L-P). Each point represents an individual participant’s analyte measurement, with horizontal lines indicating the median and IQR. Dotted lines represent LoD, see Supplementary Table S2 for all LoD values and Supplementary Table S4 for descriptive statistics. Statistical significance tested by Kruskal-Wallis with a Dunn’s correction for multiple comparisons. ns=not significant, *p<0.05, **p<0.01, ***p<0.001, ****p<0.0001.

## Supplementary Figure S3. Immune factors with a high proportion of values below the LoD across all plasma types. Immune factors were measured using (A, C) ELISAs and (B) LEGENDplex assays in plasma collected from cord (C) blood, placental (P) blood, and at the postdelivery (PD) timepoint. Each point represents an individual participant’s analyte measurement, with horizontal lines indicating the median and IQR. Dotted lines represent LoD, see Supplementary Table S2 for all LoD values and Supplementary Table S4 for descriptive statistics. Statistical significance tested by Kruskal-Wallis with a Dunn’s correction for multiple comparisons. ns=not significant, *p<0.05, **p<0.01, ****p<0.0001.

## Supplementary Figure S4. Soluble factor expression in cord, placental, and postdelivery plasma. Soluble factors were measured using (A-B, I-J) ELISAs and (C-H, K-L) LEGENDplex assays in plasma collected from cord (C) blood, placental (P) blood, and at the postdelivery (PD) timepoint. Immune factors were graphed in pg/ml (A-B) or ng/ml (C-L). Each point represents an individual participant’s analyte measurement, with horizontal lines indicating the median and IQR. Dotted lines represent LoD, see Supplementary Table S2 for all LoD values and Supplementary Table S4 for descriptive statistics. Statistical significance tested by Kruskal-Wallis with a Dunn’s correction for multiple comparisons. ns=not significant, *p<0.05, **p<0.01, ***p<0.001, ****p<0.0001.

## Supplementary Figure S5. Correlations between immune factors in cord blood plasma. Graphical representation of two-tailed Spearman correlations between immune factors in fetal cord blood plasma. Spearman r and p-values are in Supplementary Table S8.

## Supplementary Figure S6. Correlations between immune factors in placental plasma. Graphical representation of two-tailed Spearman correlations between immune factors in placental plasma. Spearman r and p-values are in Supplementary Table S9.

## Supplementary Figure S7. Immune factors that significantly correlate with gestational age at delivery. Log_2_ concentrations of immune factors were plotted against the gestational age at delivery in weeks. Each dot represents an individual participant’s analyte measurement. Statistical significance tested by Spearman correlation (r_S_) and approximate p-value of the correlation displayed. Only those with a p-value<0.05 and an r_S_>0.2 or <-0.2 were considered significant.

## Supplementary Figure S7 continued. Immune factors that significantly correlate with gestational age at delivery. Log_2_ concentrations of immune factors were plotted against the gestational age at delivery in weeks. Each dot represents an individual participant’s analyte measurement. Statistical significance tested by Spearman correlation (r_S_) and approximate p-value of the correlation displayed. Only those with a p-value<0.05 and an r_S_>0.2 or <-0.2 were considered significant.

## Supplementary Figure S8. Immune factors associated with preterm birth. Immune factor concentrations were measured in plasma collected from participants at enrollment, postdelivery, and postpartum timepoints, as well as from placenta and cord blood. Concentrations were compared between individuals who delivered at term (T, n = 83) and preterm births (PTB, n = 35). Each point represents an individual participant’s analyte concentration; horizontal lines indicate the median and IQR. Statistical significance was assessed using a two-tailed Mann–Whitney test. *p<0.05, ns signifies a p value between 0.05 and 0.1. See Supplementary Table S5 for descriptive statistics.

## Supplementary Figure S9. Trimester-specific immune factor concentrations in term versus preterm birth. Individuals were grouped by their enrollment in the second or third trimester. Immune factors were assessed for significance by trimester, and concentrations were compared between term and preterm births within each specific trimester. Panels (A, C, E, G, I, K, M, O) display all immune factors that varied significantly by trimester, showed a significant (p < 0.05) or trending (0.05 < p < 0.1) association with preterm birth at enrollment (Fig. 2, Supplementary Fig. S8), or were significantly associated with preterm birth within any single trimester. Panels (B, D, F, H, J, L, N, P) compare individuals with a term birth (T) who enrolled in the second (n=33) or third (n=50) trimester against those with a preterm birth (PTB) who enrolled in the second (n=21) or third (n=14) trimester. Statistical significance was evaluated using a two-tailed Mann-Whitney U test (A, C, E, G, I, K, M, O) or a Kruskal-Wallis test uncorrected for multiple comparisons (B, D, F, H, J, L, N, P).

## Supplementary Figure S9 continued. Trimester-specific immune factor concentrations in term versus preterm birth. Individuals were grouped by their enrollment in the second or third trimester. Immune factors were assessed for significance by trimester, and concentrations were compared between term and preterm births within each specific trimester. Panels (A, C) display all immune factors that varied significantly by trimester, showed a significant (p < 0.05) or trending (0.05 < p < 0.1) association with preterm birth at enrollment (Fig. 2, Supplementary Fig. S8), or were significantly associated with preterm birth within any single trimester. Panels (B, D) compare individuals with a term birth (T) who enrolled in the second (n=33) or third (n=50) trimester against those with a preterm birth (PTB) who enrolled in the second (n=21) or third (n=14) trimester. Statistical significance was evaluated using a two-tailed Mann-Whitney U test (A, C) or a Kruskal-Wallis test uncorrected for multiple comparisons (B, D).

## Supplementary Figure S10. Immune factors associated with perinatal death. Immune factor concentrations were measured in plasma collected from participants at enrollment and postdelivery timepoints, as well as from placenta and cord blood. Concentrations were compared between live births (LB, n = 108) and perinatal deaths (PND, n = 10). Each point represents an individual participant’s analyte concentration; horizontal lines indicate the median and IQR. Statistical significance was assessed using a two-tailed Mann–Whitney test. *p<0.05, **p<0.01, ***p<0.001, ns signifies a p value between 0.05 and 0.1. See Supplementary Table S6 for descriptive statistics.

## Supplementary Figure S10 continued. Immune factors associated with perinatal death. Immune factor concentrations were measured in plasma collected from participants at postdelivery and postpartum timepoints. Concentrations were compared between live births (LB, n = 108) and perinatal deaths (PND, n = 10). Each point represents an individual participant’s analyte concentration; horizontal lines indicate the median and IQR. Statistical significance was assessed using a two-tailed Mann–Whitney test. *p<0.05, **p<0.01, ns signifies a p value between 0.05 and 0.1. See Supplementary Table S6 for descriptive statistics.

## Supplementary Figure S11. Trimester-specific immune factor concentrations in live birth versus perinatal death. Panels (A, C, E, G, I, K, M, O) display all immune factors that varied significantly by trimester, showed a significant (p < 0.05) or trending (0.05 < p < 0.1) association with perinatal death at enrollment (Fig. 3, Supplementary Fig. S10, S10 continued), or were significantly associated with perinatal death within any single trimester. Panels (B, D, F, H, J, L, N, P) compare individuals with a subsequent live birth (LB) who enrolled in the second (n=51) or third (n=57) trimester against those who experienced a stillbirth or neonatal death (PND) and enrolled in the second (n=3) or third (n=7) trimester. Statistical significance was evaluated using a two-tailed Mann-Whitney U test (A, C, E, G, I, K, M, O) or a Kruskal-Wallis test uncorrected for multiple comparisons (B, D, F, H, J, L, N, P).

## Supplementary Figure S12. Immune factors associated with low birthweight. Immune factor concentrations were measured in plasma collected from participants at enrollment and postpartum timepoints, as well as from cord blood. Concentrations of analytes were compared between individuals with normal birthweight (N, n = 99), low birthweight (LBW, n = 9), and those with missing birthweight data (M, n = 10). Each point represents an individual participant’s analyte concentration; horizontal lines indicate the median and IQR. Statistical significance tested by Kruskal-Wallis uncorrected for multiple comparison. *p<0.05, p-values between 0.05 and 0.1 labeled, ns=p-value>0.1. See Supplementary Table S7 for descriptive statistics.

## Supplementary Figure S13. Immune factors that significantly correlate with birthweight. Log_2_ concentrations of immune factors were plotted against the birthweight in kilograms. Each dot represents an individual participant’s analyte measurement. Statistical significance tested by Spearman correlation (r_S_) and approximate p-value of the correlation displayed. Only those with a p-value<0.05 and an r_S_>0.2 or <-0.2 were considered significant. Horizontal dotted lines represent the point at which y=0.

## Supplementary Figure S14. Trimester-specific immune factor concentrations in normal versus low birthweight. Individuals were grouped by their enrollment in the second or third trimester. Immune factors were assessed for significance by trimester, and concentrations were compared between normal (N) and low birthweight (LBW) neonates within each specific trimester. Panels (A, C, E, G, I, K, M, O) display all immune factors that varied significantly by trimester, showed a significant (p < 0.05) or trending (0.05 < p < 0.1) association with low birthweight at enrollment (Fig. 4, Supplementary Fig. S12), or were significantly associated with low birthweight within any single trimester. Panels (B, D, F, H, J, L, N, P) compare individuals who gave birth to normal birthweight neonates and enrolled in the second (n=44) or third (n=55) trimester against those who gave birth to low birthweight neonates and enrolled in the second (n=2) or third (n=7) trimester. Statistical significance was evaluated using a two-tailed Mann-Whitney U test (A, C, E, G, I, K, M, O) or a Kruskal-Wallis test uncorrected for multiple comparisons (B, D, F, H, J, L, N, P).

## Supplementary Figure S14 continued. Trimester-specific immune factor concentrations in normal versus low birthweight. Individuals were grouped by their enrollment in the second or third trimester. Immune factors were assessed for significance by trimester, and concentrations were compared between normal (N) and low birthweight (LBW) neonates within each specific trimester. Panels (A, C, E) display all immune factors that varied significantly by trimester, showed a significant (p < 0.05) or trending (0.05 < p < 0.1) association with low birthweight at enrollment (Fig. 4, Supplementary Fig. S12), or were significantly associated with low birthweight within any single trimester. Panels (B, D, F) compare individuals who gave birth to normal birthweight neonates and enrolled in the second (n=44) or third (n=55) trimester against those who gave birth to low birthweight neonates and enrolled in the second (n=2) or third (n=7) trimester. Statistical significance was evaluated using a two-tailed Mann-Whitney U test (A, C, E) or a Kruskal-Wallis test uncorrected for multiple comparisons (B, D, F).

## Supplementary Figure S15. Composite inflammatory score across birth outcomes. Median inflammatory scores for no known adverse outcome (None), preterm birth (PTB), perinatal death (PND), and low birthweight (LBW), summed across plasma types. Scores were derived from 26 pro-inflammatory and 2 anti-inflammatory factors using the 10^th^ and 90^th^ percentiles (Supplementary Table S3). Positive values indicate a more inflammatory profile. Participants can belong to multiple groups; individuals missing birthweight with no other adverse outcome were excluded.

## Supplementary Figure S16. Comparison of immune factors based on placental malaria status. Levels of immune factors in placental plasma were analyzed across four groups defined by placental malaria status: none observed (No, n=2), active (A, n=6), active-chronic (AC, n=12), and past-chronic (PC, n=81). Three subjects lacking placental malaria data were excluded from this analysis. Significance was determined using a Kruskal-Wallis test, uncorrected for multiple comparisons. The figure displays only those immune factors demonstrating statistical significance or a p-value (0.05 < p < 0.1). Significance levels are denoted as *p < 0.05 and **p < 0.01.
